# Module and individual domain deletions of NRPS to produce plipastatin derivatives in *Bacillus subtilis*

**DOI:** 10.1186/s12934-018-0929-4

**Published:** 2018-05-31

**Authors:** Ling Gao, Jianping Guo, Yun Fan, Zhi Ma, Zhaoxin Lu, Chong Zhang, Haizhen Zhao, Xiaomei Bie

**Affiliations:** College of Food Science and Technology, Nanjing Agricultural University, 1 Weigang, Nanjing, 210095 People’s Republic of China

**Keywords:** Genetic engineering, Plipastatin synthetase, Novel lipopeptides, Module skipping

## Abstract

**Background:**

Plipastatin, an antifungal lipopeptide, is synthesized by a non-ribosomal peptide synthetase (NRPS) in *Bacillus subtilis*. However, little information is available on the combinatorial biosynthesis strategies applied in plipastatin biosynthetic pathway. In this study, we applied module or individual domain deletion strategies to engineer the plipastatin biosynthetic pathway, and investigated the effect of deletions on the plipastatin assembly line, as well as revealed the synthetic patterns of novel lipopeptides.

**Results:**

Module deletion inactivated the entire enzyme complex, whereas individual domain (A/T domain) deletion within module 7 truncated the assembly line, resulting in truncated linear hexapeptides (C_16~17_β-OHFA-Glu-Orn-Tyr-Thr-Glu-Ala/Val). Interestingly, within the module 6 catalytic unit, the effect of thiolation domain deletion differed from that of adenylation deletion. Absence of the T_6_-domain resulted in a nonproductive strain, whereas deletion of the A_6_-domain resulted in multiple assembly lines via module-skipping mechanism, generating three novel types of plipastatin derivatives, pentapeptides (C_16~17_β-OHFA-Glu-Orn-Tyr-Thr-Glu), hexapeptides (C_16~17_β-OHFA-Glu-Orn-Tyr-Thr-Glu-Ile), and octapeptides (C_16~17_β-OHFA-Glu-Orn-Tyr-Thr-Glu-Gln-Tyr-Ile).

**Conclusions:**

Notably, a unique module-skipping process occurred following deletion of the A_6_-domain, which has not been previously reported for engineered NRPS systems. This finding provides new insight into the lipopeptides engineering. It is of significant importance for combinatorial approaches and should be taken into consideration in engineering non-ribosomal peptide biosynthetic pathways for generating novel lipopeptides.

**Electronic supplementary material:**

The online version of this article (10.1186/s12934-018-0929-4) contains supplementary material, which is available to authorized users.

## Background

Microorganisms produce a large variety of small bioactive peptides, many of which are biosynthesised by multifunctional megasynthetases known as nonribosomal peptide synthetases (NRPSs). Prominent biosurfactants (e.g., surfactin), drugs (e.g., vancomycin and cyclosporin A) and fungicides (e.g., iturin A and fengycin) are examples of compounds derived from nonribosomal peptide biosynthesis [1–3]. Compared with the polypeptides produced by ribosomal synthesis, peptides built on NRPSs are typically shorter (up to about 20 residues), linear, cyclic or branched-cyclic, and often contain non-proteinogenic amino acids as building blocks. Amino acids can be modified by peptide synthetases through epimerisation, methylation or hydroxylation, resulting in enormous structural diversity of peptides that makes many compounds difficult to chemically synthesise [3, 4].

**Fig. 1:**
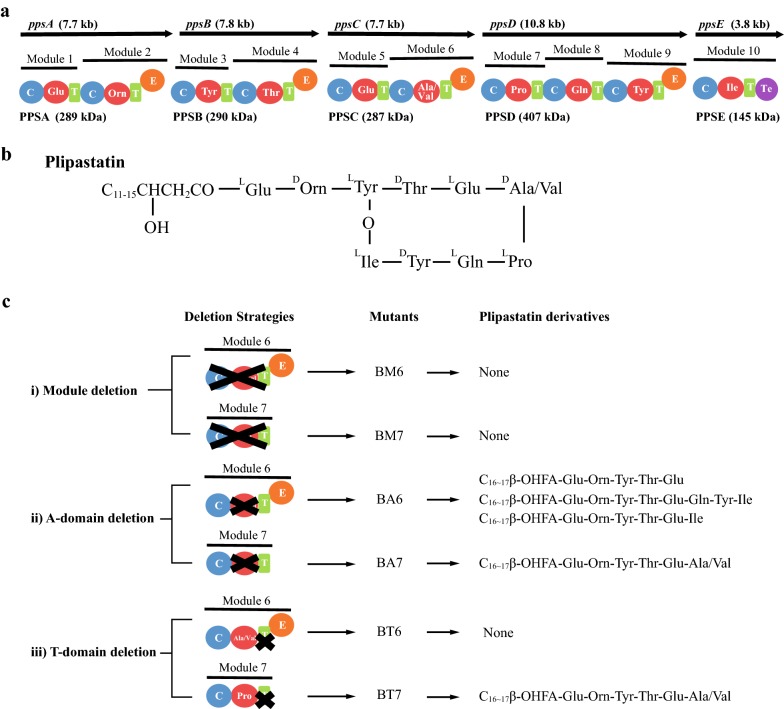
**a** Schematic diagram of the plipastatin biosynthetic system. **b** Structure of plipastatin. **c** Deletion strategies: (i) deleting module 6 or 7 in plipastatin NRPS respectively, (ii) Deleting single A_6_ or A_7_ domain, (iii) deleting single T_6_ and T_7_ domain. Plipastatin NRPS assembly line contains five subunits, encoded by five genes *ppsABCDE*. Base on their function, the plipastatin synthetase are divided into 10 modules. Each module is comprised of condensation (C), adenylation (A) and thiolation (T) domains. Module 2, 4, 6 and 9 contains an epimerization (E) domian. A thioesterase (Te) domain is located on the C-teminus of module 10, which is responsible for products cyclisation and hydrolysis

NRPSs are often composed of a series of specific amino acid-incorporating modules, each consisting of three catalytic domains [5]; condensation (C) domains couple activated amino acids to the growing peptide chain, adenylation (A) domains selectively activate specific amino acids, and thiolation (T) domains, also called peptidyl carrier proteins (PCPs), tether the activated amino acids and growing peptide chains through the cofactor 4′-phosphopantetheine. The final module usually contains an additional thioesterase (Te) domain to release products by cyclisation or hydrolysis. In the plipastatin NRPS [6–8], modules 2, 4, 6 and 9 contain an epimerase (E) domain after the T domain, which converts l-amino acids into d-amino acids (Fig. 1a). The lipopeptide plipastatin, also known as fengycin, possesses strong biological activity against phospholipase A2 and filamentous fungi [9]. The compound has a 10 amino acid core cyclised by an intramolecular ester bond to make an eight-membered ring with a two residue side chain linked with a β-hydroxy fatty acid chain (C_14_ to C_18_) at the N-terminal residue in the chain (Fig. 1b). Plipastatin is assembled on five giant NRPS multi-enzymes, PPSA, PPSB, PPSC, PPSD, and PPSE, encoded by the *ppsA, ppsB, ppsC, ppsD,* and *ppsE* genes, respectively, in the plipastatin synthetase operon (Fig. 1a).

Because NRPSs have a modular architecture, the order and specificity of modules determines the sequence of amino acid residues in the final peptide products. This organisation is conducive to straightforward strategies for constructing hybrid NRPSs in which functional modules (C-A-T or C-A-T-E) or domains are deleted and replaced to produce novel peptide analogs [10–12]. However, genetic engineering of the plipastatin synthetase complex has not been exploited for the biosynthesis of novel lipopeptides, and combinatorial biosynthesis strategies related to the plipastatin biosynthetic pathway have not been widely reported. Surfactin synthetases and daptomycin synthetases have been developed into a model system for manipulation of NRPSs, and novel compounds have been successfully produced via module or domain exchange [13–15]. Based on the crystal structure of the substrate selecting/activating A domain, the specificity-conferring codes in the active sites of A domains have been exploited to modulate specificity in NRPSs using site-specific mutagenesis and subdomain swapping [16, 17]. These successes demonstrate the potential to further explore and exploit plipastatin biosynthesis to build novel lipopeptides by genetic engineering.

We recently investigated the production of bioengineered plipastatin analogs by thioesterase domain- and inter-module communication domain-mediated reprogramming of the plipastatin NRPS complex [18, 19]. In the present study, we attempted to further modify the plipastatin amino acid core by deleting complete modules or individual domains, and subsequently evaluated the effect of these changes on the plipastatin assembly line. Additionally, we coupled the same catalytic unit with deletion of the entire module or single domains and investigated the relationships between deletions and novel lipopeptide derivatives catalysed by plipastatin synthetases.

## Methods

### Strains and culture conditions

Bacterial strains and plasmids used in this study are shown in Additional file 1: Table S1. Bacterial strains were routinely grown on Luria–Bertani (LB) agar plates or in LB broth at 37 °C. Seed medium contained 5 g L^−1^ beef extract, 10 g L^−1^ peptone, 5 g L^−1^ yeast extract, 5 g L^−1^ NaCl, and 10 g L^−1^ glucose. Fermentation medium used to produce lipopeptides was prepared as described previously [19]. *Escherichia coli* DH5α was used for general cloning, and JM110 was used for demethylation of plasmids. When required, kanamycin (50 μg mL^−1^ for *E. coli* or 20 μg mL^−1^ for *Bacillus*) was added to the culture medium.

### Construction of deletion plasmids

Deletion plasmids used for module or individual domain deletion in the plipastatin synthetase (Fig. 1) were all derivatives of pKS2. Deletions of modules 6 or 7 were performed using plasmids pKS-ΔM6 and pKS-ΔM7. Plasmids pKS-ΔA6 and pKS-ΔA7 were used to knockout the respective A-domain, while pKS-ΔT6 and pKS-ΔT7 were constructed to delete the T-domain of modules 6 or 7, respectively. All primers used are listed in Additional file 1: Table S2 and were designed with Primer 5.0 based on genes encoding plipastatin synthetase subunits C and D (Gene ID 940102 and 940013, respectively).

For pKS-ΔM6, fragments of DNA upstream and downstream of module 6 (C-A-T) were amplified by PCR with primer pairs Module6U-F/R and Module6D-F/R, respectively. The 0.75 kb upstream fragment (*ppsC*: 2365–3114 region; coordinates refer to Gene ID 940102 in the *ppsC* gene cluster) consisted of a portion of the 3′ end of the gene encoding module 5, while the 0.56 kb downstream fragment (*ppsC*: 6211–6774 region) included the gene region encoding the T-E linker and a portion of E-domain. These two fragments were used as templates for splice overlap extension polymerase chain reaction (SOE-PCR) with primers Module6SOE-F/R. A 1.3 kb fusion fragment including the upstream and downstream regions of module 6 (C-A-T) was obtained and cloned into the *Sal*I-*Kpn*I sites in pKS2 to yield pKS-ΔM6. All inserted fragments were confirmed by sequencing (Genscript, Nanjing, China). Other deletion plasmids pKS-ΔM7, pKS-ΔA6, pKS-ΔA7, pKS-ΔT6 and pKS-ΔT7 (Additional file 1: Table S1) were constructed by a similar protocol.

### Construction of *Bacillus subtilis* deletion mutants

Each deletion plasmid was introduced into *B. subtilis* pB2-L by traditional chemical transformation [20]. The pKS-based vector carries a kanamycin resistance (Kn^R^) marker, and transformants were selected at 30 °C on LB agar with 20 μg mL^−1^ Kn. The deletion mutants were selected by a two-step replacement recombination procedure as described previously [19, 21]. Growth at 37 °C, a non-permissive temperature for plasmid replication, in the presence of kanamycin selects for clones in which the plasmid has been integrated into the chromosome between the target gene and a homologous sequence on the plasmid by a single crossover. Subsequently, a separate clone of the integrant was cultured in LB medium at 30 °C for 48 h to induce a second crossover event and excise the plasmid. Kanamycin-sensitive (Kn^S^) clones with either the parental or deletion sequence were obtained and verified for genotype by PCR analysis and sequencing. All deletions maintained the linker regions between the target module/domain and adjacent domains.

### Lipopeptide production, purification, and identification

Mutant strains from LB agar plates were inoculated into 20 mL of seed medium, and the whole preculture was inoculated into 200 mL of fermentation medium in 1 L flasks and cultured at 30 °C for 3 days with shaking at 180 r min^−1^. Cultures were centrifuged at 5000×*g* for 15 min at 4 °C to remove bacterial cells, and the supernatant was adjusted to pH 2.0 with 6 M HCl to precipitate plipastatin and plipastatin-derived peptides. The pellet was then collected and extracted with methanol, and the methanol supernatant was evaporated to dryness and redissolved in 2 mL of methanol. Subsequently, the crude extract was filtered through a 0.22 μm filter and analysed by high-resolution liquid chromatography–electrospray ionisation–mass spectrometry (LC–ESI–MS) using a G2-XS Q-TOF mass spectrometer (Waters, USA). A 5 μL aliquot of crude extract was loaded onto a UPLC column (2.1 × 100 mm ACQUITY UPLC BEH C18 column containing 1.7 μm particles), and eluted with a solvent gradient of 5–95% buffer B for 22 min (buffer A = H_2_O + 0.1% formic acid; buffer B = acetonitrile + 0.1% formic acid) at a flow rate of 0.4 mL min^−1^ and monitoring at 205 nm. Mass spectrometry was performed using an electrospray source in positive ion mode within a mass range of 50–1500 m/z. Ionisation was performed with a capillary voltage of 2.5 kV, a collision energy of 40 eV, a source temperature of 120 °C, and a desolvation gas temperature of 400 °C. Data acquisition and processing were performed using Masslynx 4.1 (Waters, USA).

## Results

### Effects of module deletion on plipastatin synthetase

To study the plipastatin biosynthesis pathway with the intention of building novel lipopeptides by genetic engineering, the complete amino acid-incorporating module (C-A-T) was deleted. We knocked out module 6 (^D^Ala/Val-incorporating module) or 7 (^L^Pro-incorporating module) in *B. subtilis* pB2-L by homologous recombination, and the corresponding mutants BM6 and BM7 were generated using a markerless module deletion (Fig. 1c). The new amino acid sequence of subunit PPSC and PPSD were showed in Additional file 2: Sequence S1 and S2, which suggested that the communication mediating (COM) domain docking interaction between PPSC and PPSD was not affected by module deletion. LC–MS analysis of the fermentation extracts from deletion strains failed to detect any plipastatin analogs (data not shown), indicating that internal module 6 or 7 are essential for lipopeptide production. Thus, we further explored the effects of individual domains in module 6 and 7 by generating additional deletion mutants.

### Effects of A-domain deletion on plipastatin synthetase

Crystal structures of the PCP-C portion of the complex revealed a highly conserved fold and a V-shaped structure [22, 23]. Our previous study showed that hybrid enzymes lacking a C-domain in module 6 are also unable to produce plipastatin derivatives (data not shown), suggesting deletion of the C-domain may severely damage protein–protein interactions and the overall structural conformation of the plipastatin NRPS, rendering deletion mutants unable to synthesise lipopeptides.

**Fig. 2:**
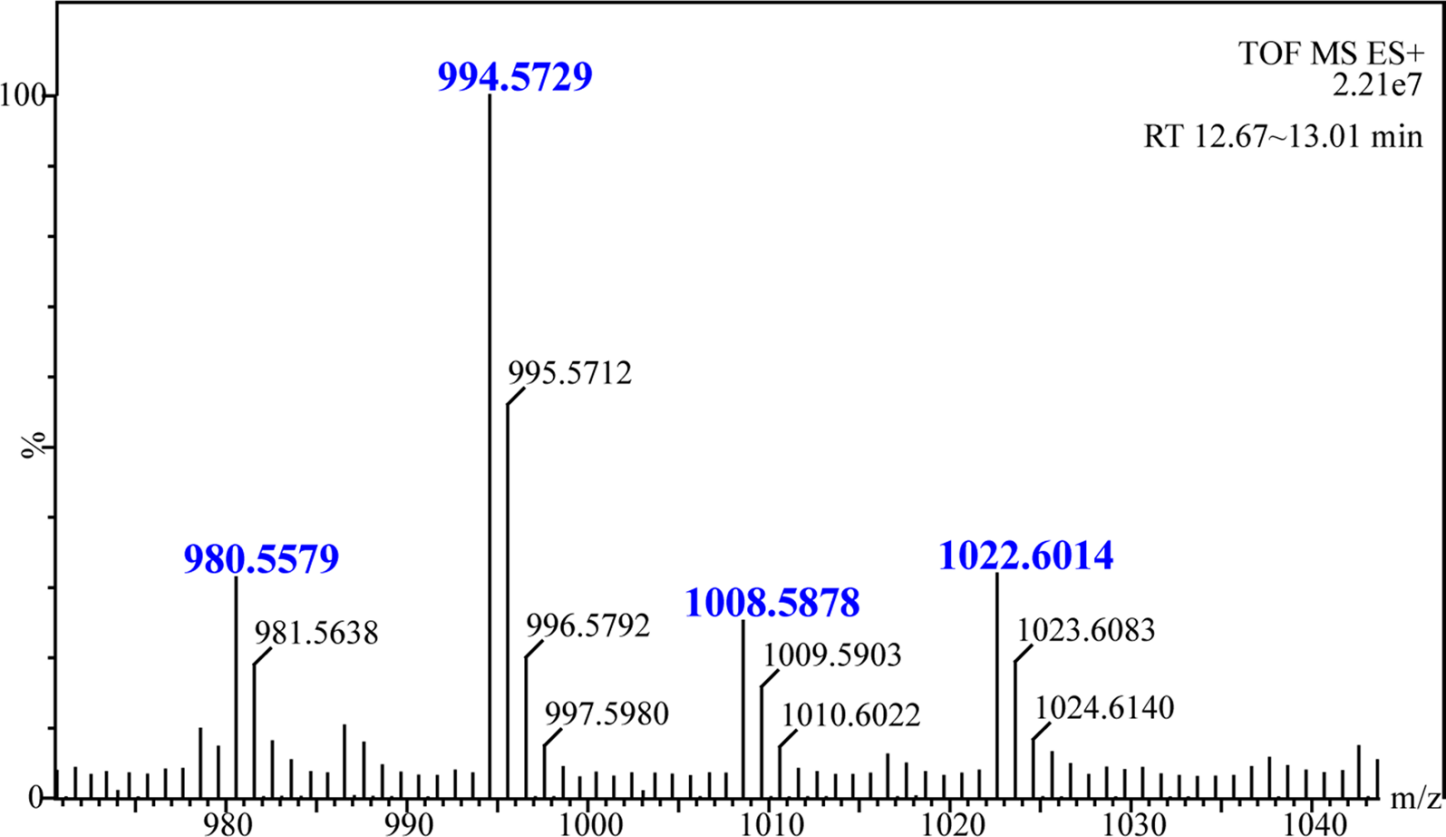
The high-resolution ESI-TOF–MS of hexapeptide ions with retention time (RT) 12.67–13.01 min produced by mutant strain BA7

**Fig. 3:**
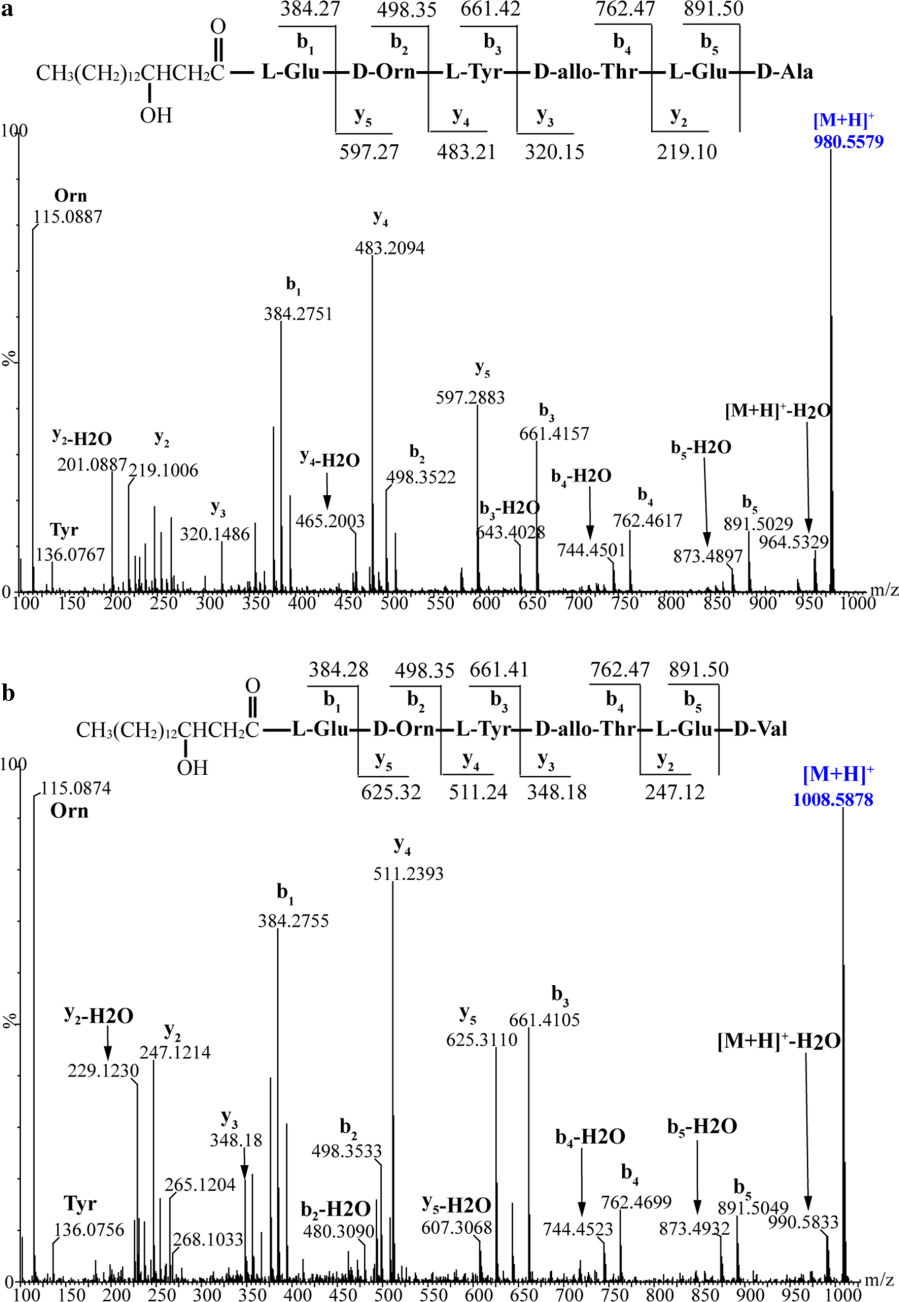
ESI-MS/MS spectra of protonated hexapeptide ions [M + H]^+^ at m/z 980.5579 **a** and m/z 1008.5878 **b**, acquired in Quadrupole-TOF (Q-TOF) mass spectrometer of crude extract from mutant strain BA7

We wondered whether deletion of the Ala/Val-activating domain (A_6_) or the Pro-activating domain (A_7_) would affect the plipastatin NRPS assembly line. The mutants BA6 (ΔA_6_) and BA7 (ΔA_7_) were constructed (Fig. 1c), and high-resolution LC–ESI–MS analysis of crude extracts from BA7 revealed a series of molecular mass ions at m/z 980.5579, 994.5729, 1008.5878, and 1022.6014 (Fig. 2), which were consistent with the mass ions of predicted truncated hexapeptides C_16~17_β-OHFA-Glu-Orn-Tyr-Thr-Glu-Ala/Val. Using MS/MS spectra of the precursor ions [M + H]^+^ at m/z 980.5579 and 1008.5878 (Fig. 3a, b), b- and y-fragment ions were assigned, which confirmed the sequence of the hexapeptide as C_16_β-OHFA-Glu-Orn-Tyr-Thr-Glu-Ala/Val. Precursor ions m/z 994.5729 and 1022.6014 with a 14 Da mass difference from m/z 980.5579 and 1008.5878 were assigned as hexapeptide variants with a C_17_β-OHFA chain. This result indicated that deletion of the A_7_ domain generated completely inactive ppsD and ppsE modules (except for the Te domain), which truncated the plipastatin NRPS complex assembly line to PPSA, PPSB, PPSC and Te domain.

**Fig. 4:**
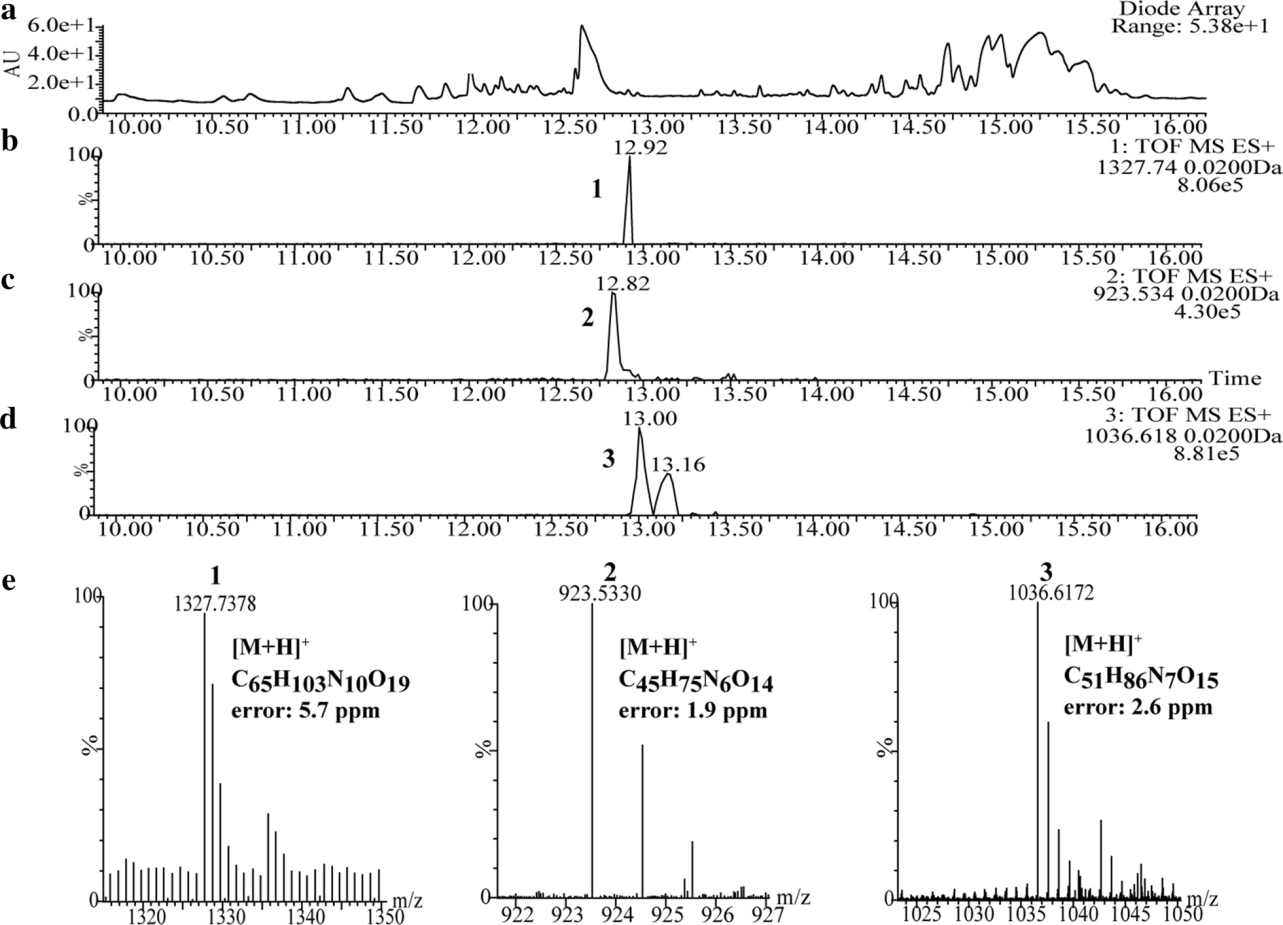
**a** LC–ESI–MS total chromatogram of crude extract from mutant strain BA6. **b** Chromatogram corresponding to m/z 1327.7401. **c** Chromatogram corresponding to m/z 923.5341. **d** Chromatogram corresponding to m/z 1036.6182. **e** High-resolution ESI–MS of parent ions from compound 1, 2 and 3

**Fig. 5:**
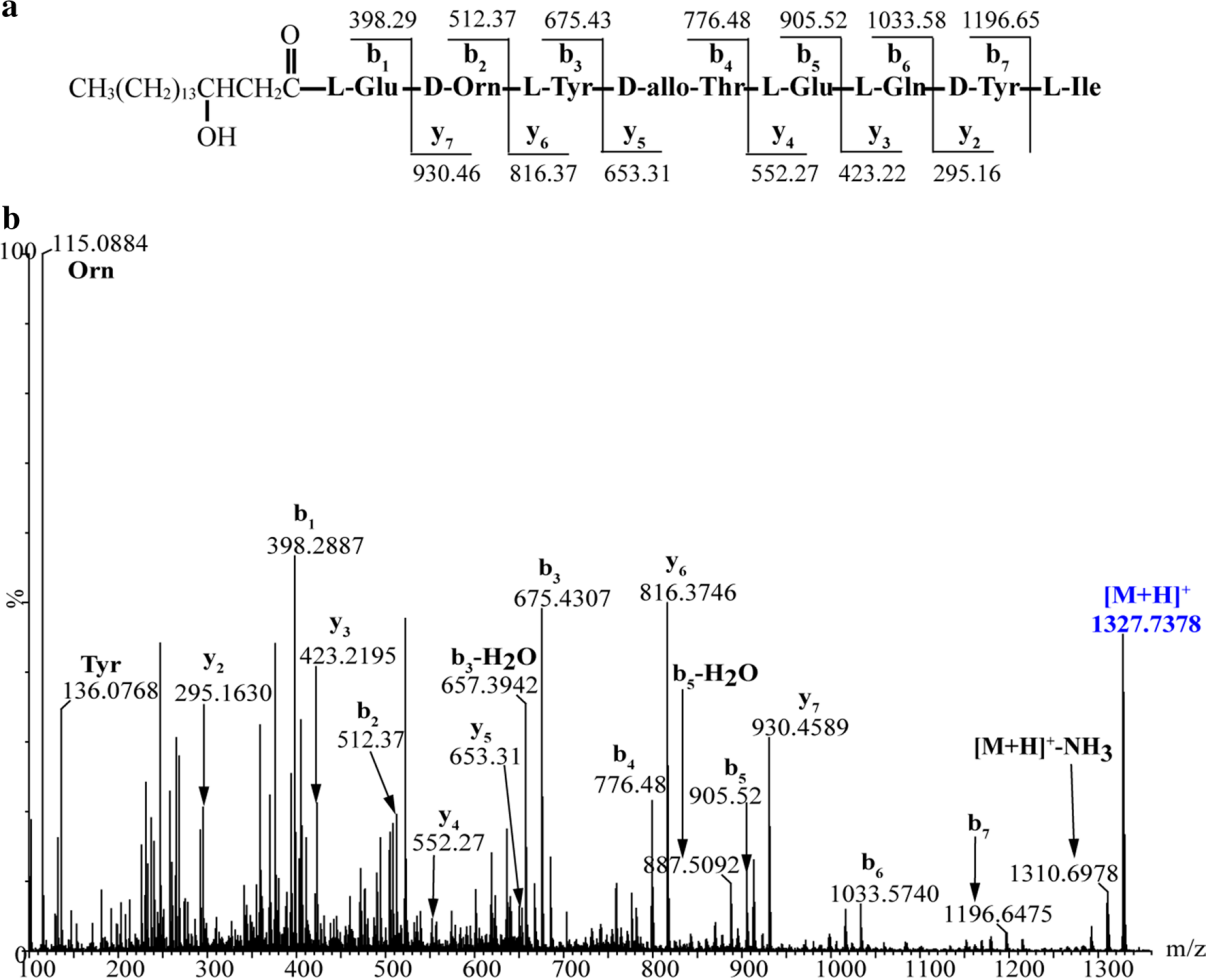
MS/MS analysis of octapeptide produced by mutant strain BA6. **a** The sequence of octapeptide and thirteen characteristic product ions b_1_ ~ b_7_ and y_2_ ~ y_7_. **b** MS/MS spectrum of octapeptide ion [M + H]^+^ at m/z 1327.7378 and assignment of key product ions

**Fig. 6:**
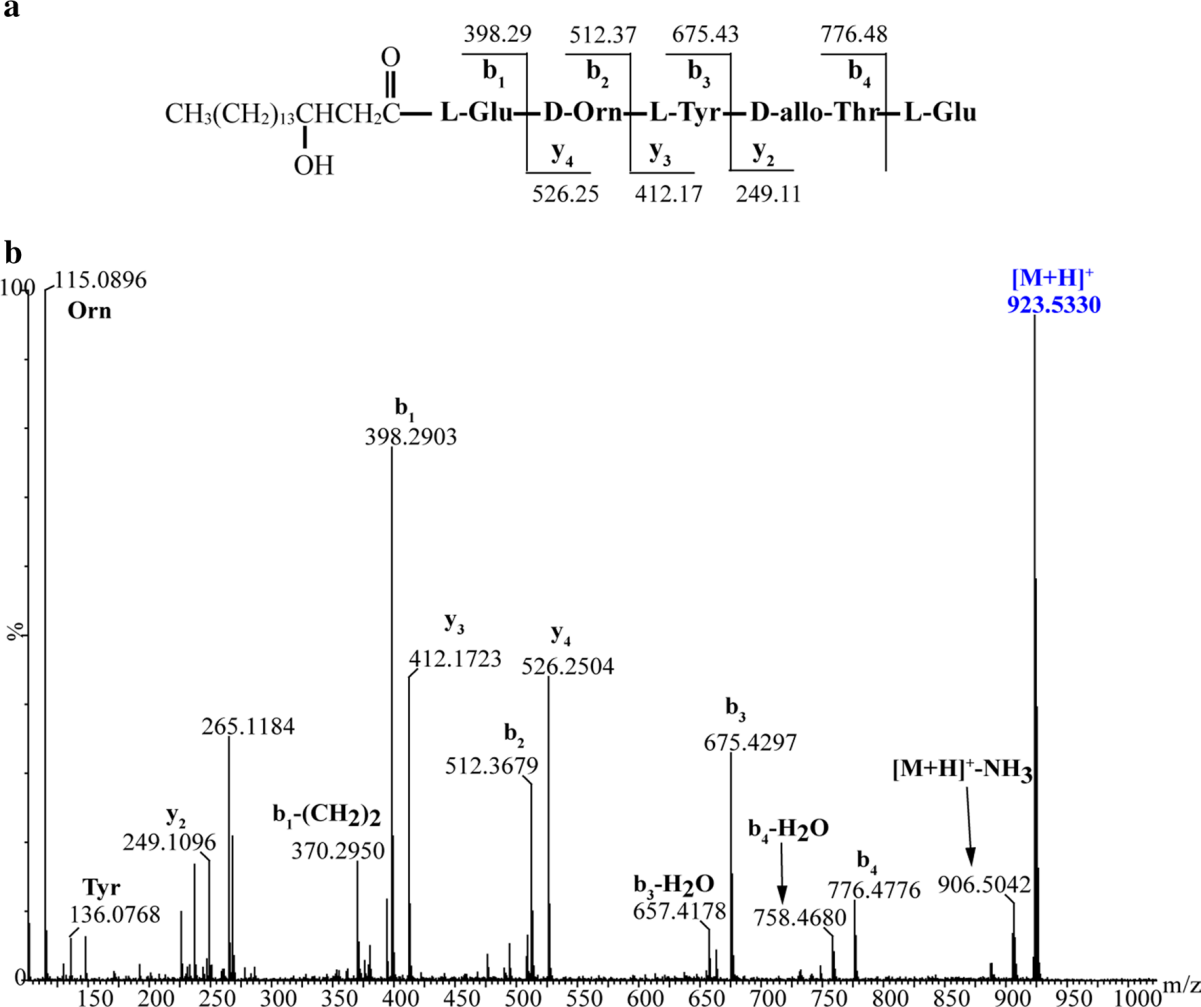
MS/MS analysis of pentapeptide produced by mutant strain BA6. **a** The sequence of pentapeptide and seven characteristic product ions b_1_ ~ b_4_ and y_2_ ~ y_4_. **b** MS/MS spectrum of pentapeptide ion [M + H]^+^ at m/z 923.5330 and assignment of key product ions

**Fig. 7:**
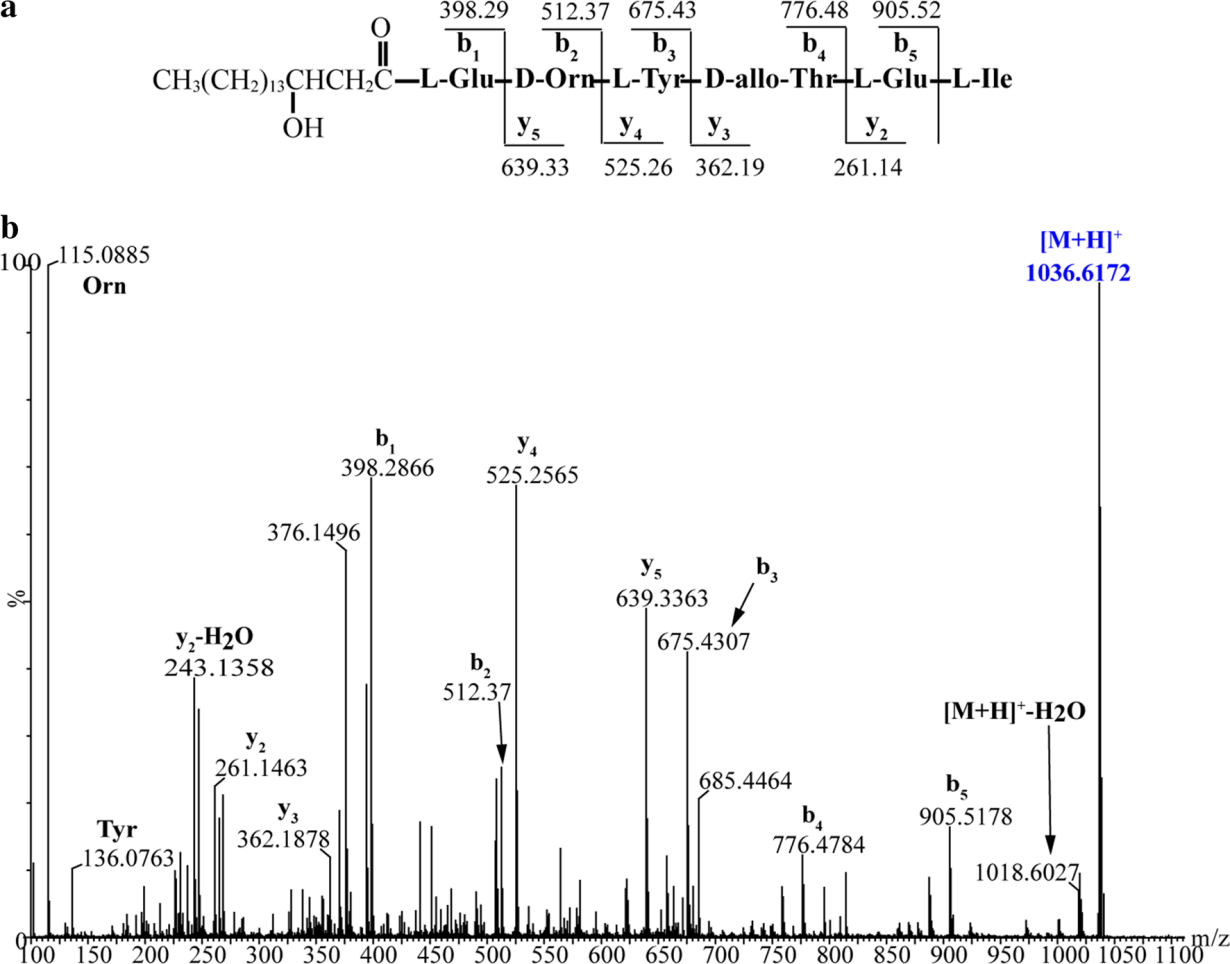
MS/MS analysis of hexapeptide produced by mutant strain BA6. **a** The sequence of hexapeptide and nine characteristic product ions b_1_ ~ b_5_ and y_2_ ~ y_5_. **b** MS/MS spectrum of hexapeptide ion [M + H]^+^ at m/z 1036.6172 and assignment of key product ions

By contrast, three types of predicted plipastatin derivatives, C_17_β-OHFA-Glu-Orn-Tyr-Thr-Glu-Gln-Tyr-Ile (**1**; m/z calcd for C_65_H_103_N_10_O_19_ [M + H]^+^ 1327.7401, found 1327.7378), C_17_β-OHFA-Glu-Orn-Tyr-Thr-Glu (**2**; m/z calcd for C_45_H_75_N_6_O_14_ [M + H]^+^ 923.5341, found 923.5330) and C_17_β-OHFA-Glu-Orn-Tyr-Thr-Glu-Ile (**3**; m/z calcd for C_51_H_86_N_7_O_15_ [M + H]^+^ 1036.6182, found 1036.6172; Fig. 4) were detected in crude extracts from mutant BA6 (ΔA_6_). These protonated molecular species of putatively assigned plipastatin derivative ions were further subjected to MS/MS analysis, and spectra of the m/z 1327.7378 ion (Fig. 5b) yielded a series of b fragment ions (m/z 1196.65 → 1033.58 → 905.52 → 776.48 → 675.43 → 512.37 → 398.29) and y fragment ions (m/z 295.16 → 423.22 → 552.27 → 653.31 → 816.37 → 930.46), as well as Orn and Tyr residues (m/z 115.09 and 136.08), consistent with hydrogen adducts of predicted octapeptides with the sequence C_17_β-OHFA-Glu-Orn-Tyr-Thr-Glu-Gln-Tyr-Ile (Fig. 5a). Similarly, MS/MS spectra of precursor ions at m/z 923.5330 and 1036.6172 (Figs. 6b, 7b) showed that all product ions were consistent with the sequences of the putative pentapeptide C_17_β-OHFA-Glu-Orn-Tyr-Thr-Glu (Fig. 6a) and hexapeptide C_17_β-OHFA-Glu-Orn -Tyr-Thr-Glu-Ile (Fig. 7a). Additionally, given the variation in the length of fatty acid chains, we also identified homologs of these plipastatin derivatives (m/z 1313.7268, 909.5298 and 1022.5976) in extracts from the BA6 mutant in which the β-OH fatty acid containing 16 carbons is linked to the N-terminus of the peptide product (Additional file 3: Figure S1). This result indicated that engineered hybrid plipastatin NRPSs lacking the A_6_-domain could form multiple assembly lines that produce plipastatin analogs.

### Effects of T-domain deletion on plipastatin synthetase

The thiolation (T) domain, also called the peptidyl carrier protein (PCP), tethers activated substrates to the growing peptide chain during plipastatin biosynthesis. To investigate the effects of deleting the T-domain and test whether deletion of the T-and A-domains in the catalytic module has the same impact, the T-domain of modules 6 and 7 were deleted, generating mutant strains BT6 (ΔT_6_) and BT7 (ΔT_7_), as outlined in Fig. 1c (strategy iii). Unlike the BA6 (ΔA_6_) mutant, BT6 was unable to produce any lipopeptides related to plipastatin, which indicated that deletion of T_6_-domain directly led to the direct inactivation of the plipastatin NRPS complex.

However, high-resolution LC–ESI–MS analysis of the crude extract from mutant BT7 revealed a series of molecular mass ions at m/z 980.5557, 994.5785, 1008.5961 and 1022.6120. Further characterisation of the putative hexapeptide was obtained from analysis of the ESI–MS/MS spectrum of [M + H]^+^ ions at 980.5557 and 1022.6120 m/z, as shown in Additional file 3: Figure S2, the results of which were consistent with the hexapeptides produced by mutant strain BA7. The LC–ESI–MS/MS data indicated that after deleting the T_7_-domain, the resulting NRPS assembly line consisting only of PPSA, PPSB, PPSC and Te domain could direct the production of the same hexapeptides generated by A_7_-domain deletion mutant.

## Discussion

Nonribosomal peptide synthetases (NRPSs) direct the production of lipopeptides via a thiotemplate mechanism [5]. An interesting feature of NRPS systems is their modular design, where individual modules act as building blocks for incorporation of single amino acid components present in the final lipopeptide product. Based on known NRPS biosynthetic principles, deletion of modules in the NRPS assembly could yield lipopeptide analogs with a decreased ring size, and a small number of successful in vivo deletions of single modules have been reported for NRPS-derived compounds. Mootz et al. [24] claimed that deleting the leucine-incorporating SrfA-A2 module in the surfactin NRPS yielded a Δ2-surfactin variant. Meanwhile, three surfactin variants (ΔLeu^3^, ΔAsp^5^, and ΔLeu^6^) were generated by knocking out modules 3, 5 and 6 of the surfactin NRPS, and the resulting ΔLeu^6^ displayed antifungal activity [25]. However, in the present study, deletion of module 6 or 7 in the plipastatin NRPS led to inactive hybrid enzymes unable to form novel NRPS assembly lines producing lipopeptides. We assume that the spatial arrangement of domains and protein interfaces in the hybrid enzyme complexes are disturbed, even though the linker regions between adjacent domains were retained in the mutant complexes. Thus, although surfactin and plipastatin are synthesised by NRPSs, the module deletion approach that worked for surfactin lipopeptide derivatives may not be suitable for the genetic modification of plipastatin synthetase. Therefore, we investigated the effects of deletion of individual domains on plipastatin synthetases.

Our previous study showed that deletion of the C-domain in module 6 inactivated the plipastatin synthetase complex. Furthermore, deletion of the T_6_-domain also inactivates the complex, preventing plipastatin production. This finding indicates that the C domain of module 6 and the T_6_-domain are likely to play an important role in the overall structural conformation of the plipastatin NRPS complex, and may be essential moieties for protein–protein interactions and efficient communication within plipastatin synthetase. By contrast, deletion of the T_7_-domain resulted in mutant complexes that could form linear hexapeptides with the sequence C_16~17_β-OHFA-Glu-Orn-Tyr-Thr-Glu-Ala/Val. Thus, we conclude that deletion of the same domain coupled with the different catalytic modules can impact the plipastatin NRPS system differently, and a similar conclusion was drawn from the deletion of the A-domain in modules 6 and 7.

In module 7 of plipastatin synthetases, the A-domain is responsible for selecting and activating the substrate proline. Based on the plipastatin biosynthetic process, deleting the A_7_-domain should generate the ΔPro^7^ plipastatin variant. However, high-resolution LC–ESI–MS/MS analysis of the crude extract from the BA7 mutant only revealed linear hexapeptides, consistent with the result of T_7_ domain deletion. This result demonstrated that deletion of the A- or T-domain of module 7 do not affect catalysis by upstream modules, but prevented downstream modules from catalysing the extension of the lipopeptide product, ultimately leading to a truncated assembly line with PPSA, PPSB, PPSC and Te domain that produce hexapeptides with the sequence C_16~17_β-OHFA-Glu-Orn-Tyr-Thr-Glu-Ala/Val.

**Fig. 8:**
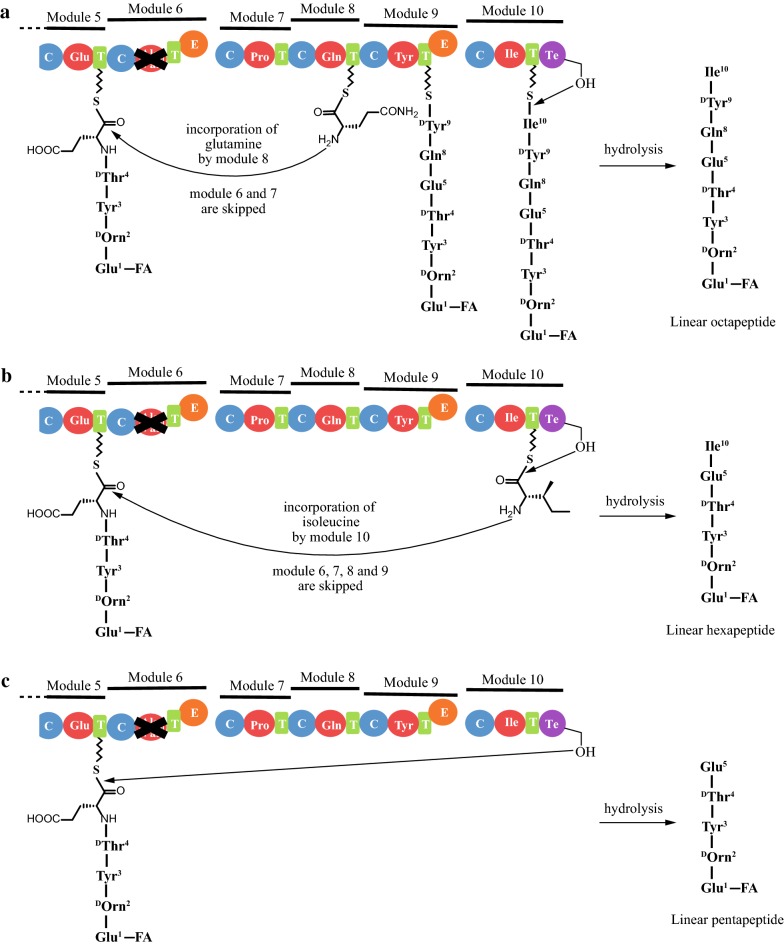
An assumed mechanism involving module skipping for mutant plipastatin NRPS without A_6_ domain. **a** Formation of linear octapeptide by complete module 6 and 7 skipping. **b** Formation of linear hexapeptide by complete module 6, 7, 8 and 9 skipping. **c** Formation of linear pentapeptide by hydrolysis in advance of thioesterase (Te) domain

Surprisingly, deletion of the A-domain of module 6, which is responsible for selecting and activating the substrate alanine or valine, generated a plipastatin hybrid enzyme complex in the BA6 mutant that produced three types of plipastatin derivatives; pentapeptides (C_16~17_β-OHFA-Glu-Orn-Tyr-Thr-Glu), hexapeptides (C_16~17_β-OHFA-Glu-Orn-Tyr-Thr-Glu -Ile), and octapeptides (C_16~17_β-OHFA-Glu-Orn-Tyr-Thr-Glu-Gln-Tyr-Ile; Fig. 1c). Comparison of the sequence of the three plipastatin derivatives revealed that they share the same five N-terminal residues (Glu-Orn-Tyr-Thr-Glu), which implies that a series of modules located upstream of module 6 retain the ability to assemble the precursor pentapeptide chain. Furthermore, we postulate a mechanism involving module skipping for mutant plipastatin NRPSs, as outlined in Fig. 8. The absence of an A_6_-domain results in a hybrid biosynthetic system that is more flexible than the native complex, that is able to incorporate the precursor pentapeptide chain with substrate Gln^8^ by skipping modules 6 and module 7, then transfer the peptide product to the T_8_-domain and continue elongation to generate octapeptides with the sequence C_16~17_β-OHFA-Glu^1^-Orn^2^-Tyr^3^-Thr^4^-Glu^5^-Gln^8^-Tyr^9^-Ile^10^), before hydrolysing and releasing the final peptide product via the thioesterase (Fig. 8a). Alternatively, incorporation with substrate Ile^10^ could occur by skipping modules 6, 7, 8, and 9, then transferring the peptide to the T-domain of module 10 to generate linear hexapeptides with the sequence C_16~17_β-OHFA-Glu^1^-Orn^2^-Tyr^3^-Thr^4^-Glu^5^-Ile^10^ (Fig. 8b). A third option involves transfer directly to the thioesterase (Te) domain to generate linear pentapeptides (C_16~17_β-OHFA-Glu^1^-Orn^2^-Tyr^3^-Thr^4^-Glu^5^; Fig. 8c). Module skipping has been described previously for hybrid polyketide synthetases [26, 27], and the skipping process was shown to involve passage of the growing polyketide through the skipped module by direct acyl carrier protein (ACP)-to-ACP transfer [27]. However, to our knowledge, module skipping phenomenon has rarely occurred in NRPS system. There was one report that module 4 was inactive and skipped completely during assembly of the myxochromide S peptide core [28]. In this context, it was unexpected that deletion of the A_6_-domain failed to generate the predicted ΔAla/Val^6^ plipastatin variant according to linear modular arrangement, but instead formed an nonconventional assembly line through skipped modules 6 and 7, or module 6, 7, 8, and 9 to produce a variety of plipastatin derivatives. This is the first report of such a process by an engineered NPRS biosynthesis pathway. This finding demonstrates that engineering NRPSs by deleting the A_6_-domain can yield diverse peptide products from complexes with greater biosynthetic potential than originally expected. An consideration in choosing module 6 for deletions was that it can recruit D-Ala or D-Val. This variability means that its adjacent domains may have more relaxed substrate specificity. It seemed to be possible to accept and transfer the altered amino acid substrates or peptide intermediates. Another consideration for choosing the module 6 and 7 is that the substrate amino acids (D-Ala/Val and L-Pro) they incorporated are location in the center of the lactone ring in plipastatin structure. We speculate that the module 6 and 7 might have a special spatial arrangement in plipastatin synthetase system. Our results demonstrated that the site of adenylation domain in module 6 is special, comparing with module 7. However, little is known about information from intramodule protein–protein interaction and substrate recognition. Whether the deletions of other A-domain located in module 2, 3, 4, and 5 will cause module-skipping, which needs to further investigations. Additionally, further protein structures of full length modules or subunits will give more detail insight into NRPS architecture for allowing a specific and efficient modification to produce novel lipopeptides.

All derivatives identified in the mutant strains were novel lipopeptides and absent from the wild-type strain. But, the original plipastatin yield only reaches 10 mg L^−1^ in *B. subtilis* pB2-L, which resulted in the production level of derivatives in these experiments were very low. The high resolution LC–MS/MS was used to detect and identify the newly derivatives from the crude extract of mutant strains. These derivatives were only detected by ion-extraction (shown in Fig. 4). It was difficult to acquire sufficient quantities of purified lipopeptides derivatives from the engineering mutant for biological testing by isolation and separation. In previous study [18], we chemically synthesized the linear plipastatin derivative (hexapeptide: C_16_β-OHFA-Glu-Orn-Tyr-Thr-Glu-Ala/Val) from company of KareBay (Ningbo, China), and tested its antimicrobial activity. The results showed that this linear hexapeptide was inactive against bacteria, but exhibited a MIC of 62.5 μg mL^−1^ against *Fusarium oxysporum*, *Rhizopus stolonifer,* and *Aspergillus ochraceus*. We think it is necessary to improve the production and purity of lipopeptides derivatives, then investigate the relationship of their structure-bioactivity in future research. In fact, similar yield reduction has been reported in daptomycin and surfactin engineering NRPS. For example, when module and subunit exchanges were used to engineer daptomycin synthetases, the production of novel antibiotics related to daptomycin generally ranged from about 1 to 50% of control [14, 15]. The yield of surfactin derivatives generated by modified the peptide synthetase were also relatively low [29–31], by module deletion was about 10% of control, by exchange of the leucine-specific domains with heterologous domains of the same specificity were about 0.1–0.5% of control [24]. Since the yields of surfactin and daptomycin could reach 1–2 g L^−1^ by optimizing fermentation parameters, the novel derivatives from engineering synthetase can be interesting for pharmaceutical applications, even though the reduction compared with the wild-type enzyme. It is possible that this approach could be applied to industrial plipastatin production for generating significant quantities of plipastatin derivatives. Besides, we might anticipate improving the yields by modification of the regulatory regions and optimizing fermentation parameters.

## Conclusions

In conclusion, deletion of modules and individual domains were used to engineer plipastatin synthetases in *B. subtilis*, leading to the production of lipopeptide analogs. All analogs identified in the mutant strains were novel lipopeptides and absent from the wild-type strain. Since none of the products was synthesised as expected. Some synthetic patterns of novel lipopeptides catalyzed by engineered NRPS have been found. Deletion of modules resulted in functionally impaired synthetase complexes, revealing that the module deletion strategy may not be suitable for engineering plipastatin synthetase to produce novel plipastatin derivatives. By contrast, deletion of individual A-/T-domains did not affect the assembly capacity of upstream modules or the hydrolytic activity of the downstream thioesterase, which led to a truncated plipastatin assembly line that produced truncated linear lipopeptides. Interestingly, a unique module-skipping process occurred following deletion of the A_6_-domain, which has not been previously reported for engineered NRPS systems. This finding suggests hybrid enzyme complexes were more flexible and diverse in terms of biosynthetic potential than envisaged based on their design. This makes it difficult to design and produce lipopeptide analogs based simply on modular sequences. But, this module skipping phenomenon offers great potential for introducing further structural diversity into nonribosomal peptides through combinatorial approaches, and should be taken into consideration when engineering NRPS biosynthetic pathways. On the other hand, engineering of NRPS to generate new and functional derivatives is extremely difficult, and few successful cases have reported in last 20 years. In our study, we successfully obtained five kinds of plipastatin derivatives through deleting module or individual domain. Comparing with this two deletion strategies, deletion of individual domains is more effective approach to generate novel plipastatin analogs, which would provide a valuable reference for exploiting combinatorial biosynthesis of NRPS to produce novel lipopeptides.

## Additional files

**Additional file 1: Table S1.** Strains and plasmids in this study. **Table S2.** PCR primers used for genetic constructs.

**Additional file 2: Sequence S1.** The new amino acid sequence of subunit PPSC. **Sequence S2.** The new amino acid sequence of subunit PPSD.

**Additional file 3: Figure S1.** ESI–MS/MS spectra of protonated ions [M + H]^+^ at m/z 1313.7268 (A), m/z 909.5298 (B) and m/z 1022.5976 (C), acquired in Quadrupole-TOF (Q-TOF) mass spectrometer of crude extract isolated from mutant strain BA6. **Figure S2.** (A) The high-resolution ESI-TOF–MS of hexapeptide ions with retention time (RT) 12.57 ~ 12.98 min from crude extract of mutant BT7. ESI–MS/MS spectra of protonated hexapeptide ions [M + H]^+^ at m/z 980.5579 (B) and m/z 1022.6120 (C), acquired in Quadrupole-TOF (Q-TOF) mass spectrometer.

## Abbreviations

NRPS: non-ribosomal peptide synthetase
C-domain: condensation domain
A-domain: adenylation domain
T-domain: thiolation domain
PCP-domain: peptidyl carrier protein
E-domain: epimerization domain
Te-domain: thioesterase domain
COM-doamin: communication mediating domain
FA: fatty acid
LC–ESI–MS/MS: liquid chromatography–electrospray ionisation–mass spectrometry/mass spectrometry
