# Supplementary material for: Module and individual domain deletions of NRPS to produce plipastatin derivatives in Bacillus subtilis
Source: Microb Cell Fact. 2018 May 31;17:84. doi: 10.1186/s12934-018-0929-4 (PMC5984369; doi:10.1186/s12934-018-0929-4)
Supplement: Supplementary file 1 — Additional file 1: Table S1. Strains and plasmids in this study. Table S2. PCR primers used for genetic constructs. [file 12934_2018_929_MOESM1_ESM.doc]

**Additional file 1: Table S1**. Strains and plasmids

| **Strain or plasmid** | **Relevant characteristic *^a^*** | **Reference or source** |
| --- | --- | --- |
| *Bacillus subtilis* |  |  |
| pB2-L | *B.subtilis* pB2 derivatives; co-producing plipastain (Pps^+^) and surfactin (Srf^+^); Cm^R^ |  |
| BM6 | pB2-L derivative without module 6 of plipastatin synthetase; Pps– Srf^+^ Cm^R^ | This study |
| BM7 | pB2-L derivative without module 7 of plipastatin synthetase; Pps– Srf^+^ Cm^R^ | This study |
| BA6 | pB2-L derivative without the 6^th^ A-domain; producing pentapeptide, hexapeptide and octapeptide; Srf^+^ Cm^R^ | This study |
| BA7 | pB2-L derivative without the 7^th^ A-domain; producing hexapeptide; Srf^+^ Cm^R^ | This study |
| BT6 | pB2-L derivative without the 6^th^ T-domain; Pps– Srf^+^ Cm^R^ | This study |
| BT7 | pB2-L derivative without the 7^th^ T-domain; producing hexapeptide; Srf^+^ Cm^R^ | This study |
| *E.coli* |  |  |
| DH5α | *recA1*, *endA1*, *lacZ*ΔM15 | Vazyme (Nanjing,China) |
| JM110 | F',*traD36* *proA^+^B^+^* *lacI^q^* *lacZ*ΔM15/*dam* *dcm* *supE44* *hsdR17* *thi leu thr rpsL lacY galK galT ara tonA tsx*Δ (lac-proAB) | Transgen Biolabs (Beijing, China) |
| Plasmids |  |  |
| pMD19T-simple | TA cloning vector; Ap^R^ | TaKaRa (Dalian, China) |
| pKS2 | Thermosensitive vector; Kan^R^, Erm^R^ |  |
| pKS-△M6 | pKS2 carrying the upstream and downstream of module 6, Kan^R^, Erm^R^ | This study |
| pKS-△M7 | pKS2 carrying the upstream and downstream of module 7, Kan^R^, Erm^R^ | This study |
| pKS-△A6 | pKS2 carrying the upstream and downstream of 6^th^ A-domain, Kan^R^, Erm^R^ | This study |
| pKS-△A7 | pKS2 carrying the upstream and downstream of 7^th^ A-domain, Kan^R^, Erm^R^ | This study |
| pKS-△T6 | pKS2 carrying the upstream and downstream of 6^th^ T-domain, Kan^R^, Erm^R^ | This study |
| pKS-△T7 | pKS2 carrying the upstream and downstream of 7^th^ T-domain, Kan^R^, Erm^R^ | This study |

***^a^*** Cm^R^, Ap^R^, Kan^R^, Erm^R^: resistant to chloramphenicol, ampicillin, kanamycin, and erythromycin, respectively.

Srf– Pps–: unable to synthesize either surfactin or plipastatin.

**Additional file 1: Table S2**. PCR primers used for genetic constructs

| **Primers** | **The sequence (5′ to 3′)** | **Restriction sites** |
| --- | --- | --- |
| Construction for module deletion | | |
| Module6U-F | GTCGACGCGCGTTTGTATGTTTTAGA | *Sal*I |
| Module6U-R | TTCAGCGGGCTTAATCGC |  |
| Module6D-F | CCATTTATCCGTAAGTCAG |  |
| Module6D-R | GGTACCCTGGTAGGAATCCGTTTTAG | *Kpn*I |
| Module6SOE-F | GCGATTAAGCCCGCTGAACCATTTATCCGTAAGTCAG |  |
| Module6SOE-R | CTGACTTACGGATAAATGGTTCAGCGGGCTTAATCGC |  |
| Module7U-F | GTCGACGGAGGAAGCAAGCTCCGTATC | *Sal*I |
| Module7U-R | AGACAGCGGATAAATATCCTGAATTGAATTCGC |  |
| Module7D-F | TTAATTATTCGAGAGGC |  |
| Module7D-R | GGTACCTTCTCGGATTAGGAGCTGAACGG | *Kpn*I |
| Module7SOE-F | GCGAATTCAATTCAGGATATTTATCCGCTGTCTTTAATTATTCGAGAGGC |  |
| Module7SOE-R | GCCTCTCGAATAATTAAAGACAGCGGATAAATATCCTGAATTGAATTCGC |  |
| Construction for A-domain deletion | | |
| A6U-F | GTCGACGCAGCTTACACCGCGTTCTTGT | *Sal*I |
| A6U-R | TGTTTGTTCTGCCTGCATTTC |  |
| A6D--F | CCTGAACCAGATGTCGCATCTAAAC |  |
| A6D-R | GGTACCTTCTACACTTCGCCAATACGCCTC | *Kpn*I |
| A6SOE-F | GAAATGCAGGCAGAACAAACACCTGAACCAGATGTCGCATCTAAAC |  |
| A6SOE-R | GTTTAGATGCGACATCTGGTTCAGGTGTTTGTTCTGCCTGCATTTC |  |
| A7U-F | GTCGACATGACGCCGTATTCGGTTCTG | *Sal*I |
| A7U-R | TGTCTTTTCCGCTTGCTTTTC |  |
| A7D-F | CCGGAGCCAGATGCTTCAATC |  |
| A7D-R | GGTACCCCTTCCAGTTCTAAGACCGCA | *Kpn*I |
| A7SOE-F | GAAAAGCAAGCGGAAAAGACACCGGAGCCAGATGCTTCAATC |  |
| A7SOE-R | GATTGAAGCATCTGGCTCCGGTGTCTTTTCCGCTTGCTTTTC |  |
| Construction for T-domain deletion | | |
| T6U-F | GTCGACCGATCGGGCGGCCGGTTGGTAAT | *Sal*I |
| T6U-R | TTCAAGTTCGTTGCGTGGCGGGATG |  |
| T6D-F | CCATTTATCCGTAAGTCAGAGAG |  |
| T6D-R | GGTACCCCTGGTAGGAATCCGTTTTAG | *Kpn*I |
| T6SOE-F | CATCCCGCCACGCAACGAACTTGAACCATTTATCCGTAAGTCAGAGAG |  |
| T6SOE-R | CTCTCTGACTTACGGATAAATGGTTCAAGTTCGTTGCGTGGCGGGATG |  |
| T7U-F | GTCGACATCCTGGAGACCCTATTCCTGAG | *Sal*I |
| T7U-R | CTCTAACAGATTTCGAGGGGCAG |  |
| T7D-F | TTAATTATTCGAGAGGC |  |
| T7D-R | GGTACCTAACCGAGACAAAAAGGC | *Kpn*I |
| T7SOE-F | CTGCCCCTCGAAATCTGTTAGAGTTAATTATTCGAGAGGC |  |
| T7SOE-R | GCCTCTCGAATAATTAACTCTAACAGATTTCGAGGGGCAG |  |

**References**

[1] Gao, L., Han, J., Liu, H., Qu, X.*, et al.*, Plipastatin and surfactin coproduction by Bacillus subtilis pB2-L and their effects on microorganisms. *Antonie van Leeuwenhoek* 2017, 1-12.

[2] Zakataeva, N. P., Nikitina, O. V., Gronskiy, S. V., Romanenkov, D. V., Livshits, V. A., A simple method to introduce marker-free genetic modifications into the chromosome of naturally nontransformable Bacillus amyloliquefaciens strains. *Appl Microbiol Biot* 2010, *85*, 1201-1209.

[3] Yang, H.-l., Wang, K., Liao, Y.-l., Wang, B.*, et al.*, Knockout of *ptsGHI* Gene of *Bacillus amyloliquefaciens* and Growth Characteristics of Corresponding Deficient Strain. *Journal of South China University of Technology (Natural Science Edition)* 2012, *40*, 95-100.
