# Supplementary material for: Module and individual domain deletions of NRPS to produce plipastatin derivatives in Bacillus subtilis
Source: Microb Cell Fact. 2018 May 31;17:84. doi: 10.1186/s12934-018-0929-4 (PMC5984369; doi:10.1186/s12934-018-0929-4)
Supplement: Supplementary file 2 — Additional file 2: Sequence S1. The new amino acid sequence of subunit PPSC. Sequence S2. The new amino acid sequence of subunit PPSD. [file 12934_2018_929_MOESM2_ESM.doc]

**Additional file 2: Sequence S1.** The new amino acid sequence of subunit PPSC. The region of Module 6 was underlined and deleted in plipastatin synthetase C (NCBI reference sequence: WP_009968965.1).

1 mpqqpeiqdi yplsfmqegm lfhslydeqs rayfeqasft ihgqldlerf qksmdavfdr

61 ydifrtafiy knvakprqvv lkqrhcpihi edishlnerd kehcteafke qdkskgfdlq

121 tdvlmrisil kwapdhyvci wshhhilmdg wclgivikdf lhiyqalgkg qlpdlppvqp

181 ygtyikwlmq qdreeaaeyw kkrlqhfeks tplpkrtdqi pngtlqqitf aipeketael

241 qkiaaasgat lntvfqalwg imlqkvnrss davfgsvisg rpselkdven mvglfintip

301 iraqsdslsf sdlvrrmqkd mneaeaysyf plydiqaqsa lkqelidhii vfentptqqe

361 ieelnqagsf dfsvkdfeme evtnyscsvk vipgrtlyvr ihfqtsayqp smmseikdyl

421 lhmvsdvisd pslpvskmtl ldedktrkiv sqnnrtvsvs peaptlhglf erqaavtper

481 lairfsggsl tyaeldmyas rlaahlaarg vtnesivgvl serspdmlia vlavlkagga

541 ylpldpaypk erlsymlkds gasllltqpg csapnfsget levdmtslec eevkrhvsas

601 vsdgslayvi ytsgstgqpk gvavehrqav sfltgmqhqf rlseddivmv ktsfsfdasv

661 wqlfwwalsg asayllppgw ekdsalivqa ihqenvttah fipamlnsfl dqaeierlsd

721 rtslkrvfag geplaprtaa rfasvlpqvs lihgygptea tvdaafyvld perdrdrlri

781 pigkpvpgar lyvldphlav qpsgvagely iagagvargy lnrpalteer fledpfylge

841 rmyktgdvar wlpdgnvefl grtddqvkir gyriepgeie aalrsiegvr eaavtvrtds

901 gepelcayve glqrnevraq lqrllpgymv paymiemeqw pvtpsgkldr nalpapggaa

961 daetyttprn vtemklsqlw edvlkngpvg ihdnffdrgg hslkatalvs riakefdvqv

1021 plkdvfahpt veglatvire gtdspyeaik paekqetypv ssaqkriyvl qqledggtgy

1081 nmpavleleg klnlermdra fkelikrhes lrtafeqdag gdpvqrihde vpftlqttvl

1141 garteeeaaa afikpfdlsq aplfraqivk vsderhlllv dmhhiisdgv svnilirefg

1201 elynnrklpa lriqykdyav wqegfktgda yktqgaywlk qlegelpvld lpadharppm

1261 rsfagdkvsf tldqevtsgl yklarengst lymvllaayt aflsrlsgqe diivgspiag

1321 rphkdlepil gmfvntlalr trpeggkpfv qylqevreta meafehqdyp feelvdklel

1381 trdmsrnplf dvmfvlqnmd qesleldelc lkpaannghq tskfdltlya qeqprglltf

1441 qmefstdlyk kktiekwlqy fnnmllsiik dnkaalgtin ilnedeahyl ihelnrtkid

1501 yprnetisrl femqaeqtpn avaivsdtqv ftyedlnswa nqiasvlqik gvgpdsvval

1561 ltgrtpelia gmlgilkagg aylpidsnlp veriaymlsd sraalllqse ktekrllgie

1621 ceqiiiediq kqgeaknves sagphslayi iytsgstgkp kgvmieqrsv irlvknsnyi

1681 tftpedrllm tssigfdvgs feifgpllng aalhlsdqqt fldshqlkry iehqgittiw

1741 ltsslfnhlt eqneqtfsql khliiggeal spshvnrirn vcpevsiwng ygptenttfs

1801 tclhiqktye lsipigrpvg nstafilnqw gvlqpvgavg elcvggdgva rgylgrpdlt

1861 kekfvphpfa pgdrlyrtgd larwlsdgti eyvgriddqv kvrgyrvelg eietalrqid

1921 gvkeaavlar taqtgskelf gyisvkagtn aeqvrsllar slpnymipay iiemetlplt

1981 sngklnrkal pepdvaskqt yipprnelee qlaliwqevl giqrigieds ffelggdsik

2041 alqvsarlgr yglslqvsdl frhpkikdls pfirkserii eqgpiqgdvp wtpvqqwffs

2101 qdieerhhfn qsvmlfhsgr lsenalrpal kklaehhdal rmvyrnddrr wiqinqgihe

2161 sqlyslrisd lsqsesgwet kikqevadlq qsinlqegpl lhaalfktlt gdylflaihh

2221 lvvdgvswri lledlsagyq qaaagqtiql ppktdsyqey arriqeyaqs sklireeayw

2281 rsveeqqaae lpyeiphhvn idfskrdsls fslteadtav llqnvnhayg tdtqdillta

2341 aslaicewtg gsklriameg hgrehilpel disrtvgwft smypalisfe nhrdelgtsv

2401 ktvkdtlgri pnkgvgygml kylthpenks itfsktpeis fnylgqfndi erqdtfrpss

2461 lgsgkditht wkreqiiems amaadkklhf nlsypparfh rntmeqlinr iehflldimk

2521 hcagqqkaek tlsdfssqsl taedldsiss lveel

COM^d^_PPSC_

**Additional file 2: Sequence S2.** The new amino acid sequence of subunit PPSD. The region of Module 7 was underlined and deleted in plipastatin synthetase D (GenBank: AIY93143.1).

1 mnmtkansiq diyplsymqe gmlfhsllqk dsqayveqas ftiegkvnpq ffqnsinalv

COM^a^_PPSD_

61 erhdifrtif isqnvsspqq vvlrernviv leedithlne aeqsqfieqw kekdrdrgfh

121 lqkdvlmria liqtgesqys ciwtfhhimm dgwclsivlk eflhiyasyv naspitlepv

181 qpygkyikwl meqdkeqavs ywdhylsghe qqtvlpkqkk tkgksrqehv tfsfskeess

241 rlselaaree vtlstifhti wgillqkynn nddavfgsvi sgrpaeiegi ehmvglfint

301 mpvrvqgakt pflqlikdmq kdrlaaeays yhplyeiqsr savkqglidh ilvfenypvq

361 qeiqmlnkqe hasdlfqihn ftvadetnys fylmvapgee ihikmnydae qhdrsfvlsv

421 kehllnavsq ilnnpnlppe eidittdtek rqligeitdq tpvyetiham fekqaektpd

481 ahavidqacs ltyrelnkaa nrlarhlrmk gvvrqepvai mmersaafit gvlgilkagg

541 aivpvdphyp adriryilhd cgcshvvsqa hlpssledny iithpedies kvdgsniksv

601 nnaddllymi ytsgttgkpk gvqfehrnma nllkfeyths gidfeadvlq fatpsfdvcy

661 qeifsallkg gtlhivpeai krdvpqlfaf inkhqtnivf lptafikmif serelansfp

721 dgvkhliaag eqlmisdlfq dvlrkrgihl hnhygpseth vvstytihpg dpipelppig

781 kpigctdlyi lnhqkqlqpc gvpgelyisg asvargyvnh dkltsdkfss dpfkpdvimy

841 rtgdlarrle dgnieyigra dnqvkirgyr iepqeievtl mnhpdiseaa iliwqdqnge

901 helcayycsv qklntidlrs ymaselpeym ipakwiwvds ipltpngkvd raalpepdas

961 isgnpytapr nlleaklsql fedvlknghi giqdnffdng ghslkatvlm sriakefhvq

1021 vslkdifahp tveglaliir eaeqnlyaai epaekrdtyp vssaqkriyv lqqldegvay

1081 nmpavleleg aldvaklsav ckelisrhep lrtsfvsgad depvqrihte vpftlskett

1141 iegfvrpfdl sqaplfragl ievsnekhvl lvdmhhiisd gvsvqllire ftdlyanrql

1201 kplriqykdy avwqqkfkkg dsyqkqetyw qqqfsgdlpi lelptdkrrp aerqfiggkv

1261 tfqldkeita rikrlahknr stlymtllal ysaflsrlsg qddivigspi agrphadlea

1321 vlgmfvntla lrtrpagnkt feeflkevrq taleayehqd ypfeelvdkl gvqremsrnp

1381 lfdttlvlqn meqqklkmnd vqlqwndleh piskfdisly vtehdselfc qfeystalfe

1441 ketiqrwasl fttlvehtaa speteldnip iltkeeerdf ieschlfeet gysmnqtlhy

1501 aleqqaektp dqaavifedg vmtykelneq anriawelig rgvkpettva iigkrspeml

1561 lgiygilkag gaylpidpdy peerisflle dsgtnilllq saglhvpeft geivylnqtn

1621 sglahrlsnp nvdvlpqsla yviytsgstg mpkgveiehr savnflnslq sryqlkhsdm

1681 imhktsysfd asiwelfwwp yagasvyllp qggekepevi akaieeqkit amhfvpsmlh

1741 aflehikyrs vpiktnrlkr vfsggeqlgt hlvsrfyell pnvsitnsyg pteatveaaf

1801 fdcpphekle ripigkpvhh vrlyllnqnq rmlpvgcige lyiagagvar gylnrpalte

1861 erfledpfyp germyktgdv arwlpdgnve flgrtddqvk irgyriepge ieaalrsieg

1921 vreaavtvrt dsgepelcay veglqrnevr aqlerllpgy mvpaymieme qwpvtpsgkl

1981 drnalpapgg aadaetytap rnvtemklsq lwedvlkngp vgihdnffdr gghslkatal

2041 vsritkefdv qvplkdvfah ptveglatvi regtdspyea ikpaekqety pvssaqkriy

2101 vlqqledggt gynmpavlel egklnpermd rafqelikrh eslrtsfeqd eggdpvqrih

2161 devpftlqtt vlgarteqea aaafikpfdl sqaplfraqi vkvsderhll lvdmhhiisd

2221 gvsvniliqe fgelynnrkl palriqykdy avwqegfktg daykmqeayw lkqlegelpv

2281 ldlpadharp pvrsfagdkv sftlepevas glhklareng stlymvllaa ytaflsrlsg

2341 qediivgspi agrphkdlep ilgmfvntla lrtrpeggkp fvqylqevre taleafehqn

2401 ypfeelvdkl eltrdmsrnp vfdamlvvqn ndyeplhlhd lqmkpaqvsh lvskfdltlq

2461 asegdgnihf lfeystalfe kttierwash ltnvlsiigk npkvtlnhid iltqeerhql

2521 lnefntgqan qygvqtisql feqqaartpk asalvsgdkt ltyqeldews ngiaralrsr

2581 gvkpdtpvgi mmhrsfsmia silgvwkagg cyvpidpeyp kerkryilsd sgtkllmtin

2641 eadlgvladf egeiltiesv eeddksplpq mssahhlayi iytsgttgrp kgvmvehkgi

2701 antlqwrrna yafnetdtil qlfsfsfdgf itsmftplls gakavllhee eakdilaikh

2761 qlsrqrithm iivpvlyral ldvvqpedvk tlrvvtlage aadreliars laicphtela

2821 neygptensv attvmrhmek qayvsigqpi dgtqvlilns nhqlqpigva gelciagtgl

2881 argyvnlpel teraftqnpf kpearmyrtg daarwmadgt leylgriddq vkirgyrvet

2941 keiesvirci kgvkdaavva hvtasgqtel sayvvtkpgl stnavrselq nklpvfmhpa

3001 fiekldslpl spngkldrga lpkpvynheg erpflppssk meqiladiwk evlgaekigt

3061 adsffelggd sikalqvsar lhrigkqmav kdlfshptiq elaayirdsd tsssqaaveg

3121 dvqwspvqkw flsqdikekh hfnqsvmlhr stsvqedalr ktlkaitchh dalrmvftqn

3181 eqgkwdqynr plshsddaly glqmidlsap dgtdgnrpye plikrhvldi qqkmdlkngp

3241 llqaglfhti dgdflflsah hlvvdgiswr vlledlalgy rqaaggedik lppktssfka

3301 yakklsdyae sqqlmkqlky wreaeeyqte alpfdqidgt rahegqrsti sftlndketa

3361 allkdansay ntdtqdmlla svilalrhwt nqsafklsle ghgredvlkg idvsrtigwf

3421 taiypllikl nadlpdsees mvhvlkttkd tlrrvpdkgf gygvikyltp pgkkdinftg

3481 apeisfnylg qfesgrtaev peedafsfsp lgaggdistt wnreqsldis aiaaegkltv

3541 nmtydnarfq rktieqlset crqfllqlie hcqnksetek tisdfddqel tedalqeiad

3601 mlsfh
