# Supplementary material for: Module and individual domain deletions of NRPS to produce plipastatin derivatives in Bacillus subtilis
Source: Microb Cell Fact. 2018 May 31;17:84. doi: 10.1186/s12934-018-0929-4 (PMC5984369; doi:10.1186/s12934-018-0929-4)
Supplement: Supplementary file 3 — Additional file 3: Figure S1. ESI–MS/MS spectra of protonated ions [M + H]+ at m/z 1313.7268 (A), m/z 909.5298 (B) and m/z 1022.5976 (C), acquired in Quadrupole-TOF (Q-TOF) mass spectrometer of crude extract isolated from mutant strain BA6. Figure S2. (A) The high-resolution ESI-TOF–MS of hexapeptide ions with retention time (RT) 12.57 ~ 12.98 min from crude extract of mutant BT7. ESI–MS/MS spectra of protonated hexapeptide ions [M + H]+ at m/z 980.5579 (B) and m/z 1022.6120 (C), acquired in Quadrupole-TOF (Q-TOF) mass spectrometer. [file 12934_2018_929_MOESM3_ESM.doc]

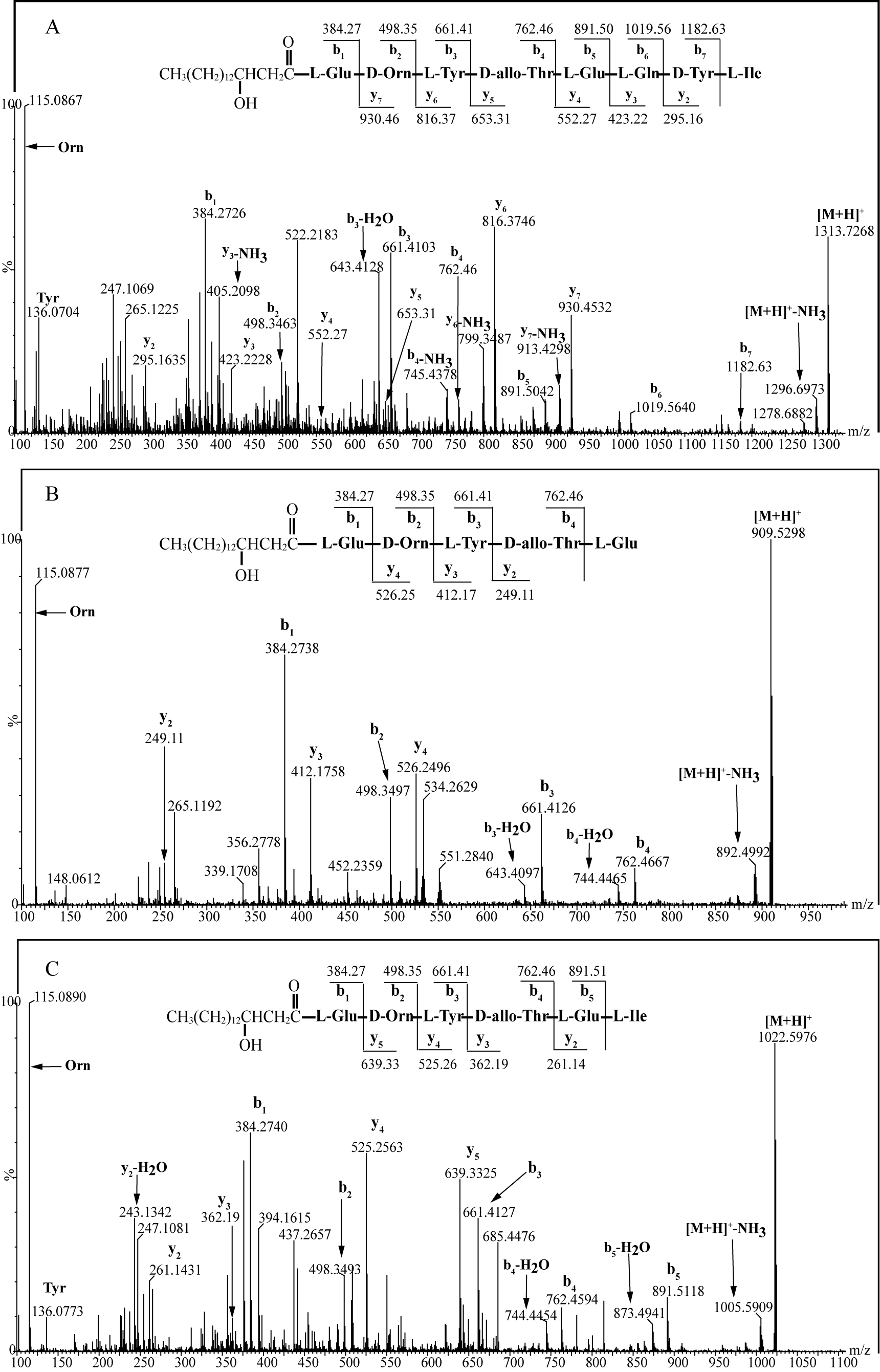


**Additional file 3: Figure S1:** ESI-MS/MS spectra of protonated ions [M+H]+ at m/z 1313.7268 (A), m/z 909.5298 (B) and m/z 1022.5976 (C), acquired in Quadrupole-TOF (Q-TOF) mass spectrometer of crude extract isolated from mutant strain BA6.


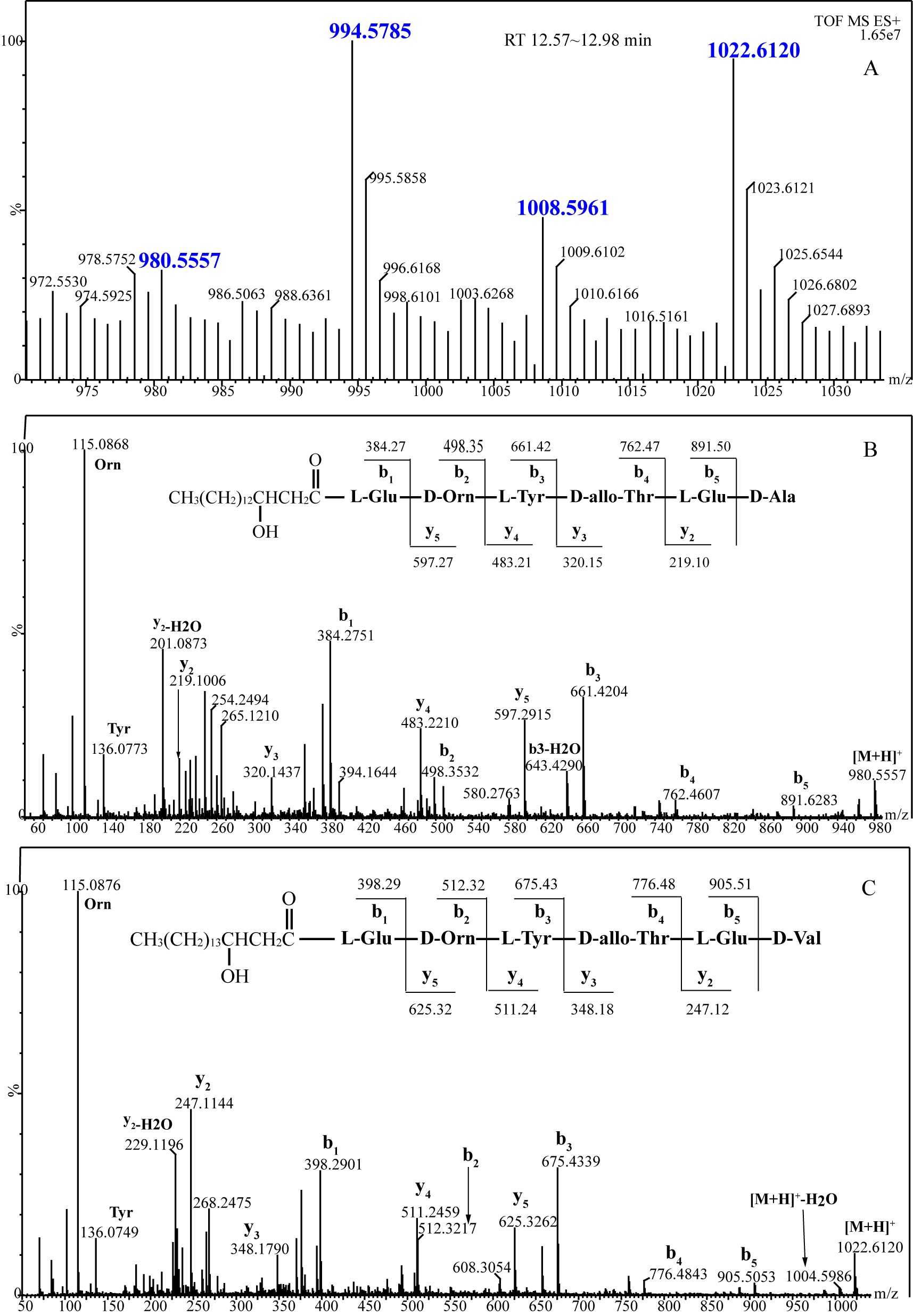


**Additional file 3: Figure S2:** The high-resolution ESI-TOF-MS of hexapeptide ions with retention time (RT) 12.57~12.98 min from crude extract of mutant BT7 (A). ESI-MS/MS spectra of protonated hexapeptide ions [M+H]^+^ at m/z 980.5579 (B) and m/z 1022.6120 (C), acquired in Quadrupole-TOF (Q-TOF) mass spectrometer.
